# Cyber Physical Systems for User Reliability Measurements in a Sharing Economy Environment

**DOI:** 10.3390/s17081868

**Published:** 2017-08-13

**Authors:** Aria Seo, Junho Jeong, Yeichang Kim

**Affiliations:** 1 Department of Techno-Management Cooperation Course, Dongguk University, 123 Dongdae-ro Gyeongju-si, Gyeongsangbuk-do 38066, Korea; seoaria@gmail.com; 2 Department of Electronic Commerce Institute, Dongguk University, 123 Dongdae-ro, Gyeongju-si, Gyeongsangbuk-do 38066, Korea; yanyenli@dongguk.edu; 3 Department of Information Management, Dongguk University, 123 Dongdae-ro, Gyeongju-si, Gyeongsangbuk-do 38066, Korea

**Keywords:** sharing economy, user reliability measurement, context-awareness computing, Internet of Things (IoT), cyber physical system (CPS)

## Abstract

As the sharing economic market grows, the number of users is also increasing but many problems arise in terms of reliability between providers and users in the processing of services. The existing methods provide shared economic systems that judge the reliability of the provider from the viewpoint of the user. In this paper, we have developed a system for establishing mutual trust between providers and users in a shared economic environment to solve existing problems. In order to implement a system that can measure and control users’ situation in a shared economic environment, we analyzed the necessary factors in a cyber physical system (CPS). In addition, a user measurement system based on a CPS structure in a sharing economic environment is implemented through analysis of the factors to consider when constructing a CPS.

## 1. Introduction

With the development of sensor networks and context-aware computing technologies, research is underway on Internet of Things (IoT) technology. The IoT is an intelligent technology and service environment that connects all objects to enable mutual communications between people and people, people and things, and things and things [1]. In an IoT environment, the computer must operate without user intervention. That is, intervention such as user control should be minimized. Recent trends in IoT technology are evolving into the notion of cyber physical systems (CPSs), which can control the physical world as well as the connections between objects. The CPS is a concept that seeks to converge with the cyber world composed of system entities, such as physical entities, sensors, actuators, and embedded systems [2].

As the era of the fourth industrial revolution began, the notion of individual ownership gradually weakened, and sharing economy begin attracting attention as a new structure for creative management. A sharing economy is an economic activity in which all the participants of a transaction can obtain mutual benefits and appropriate profits by mutually lending or exchanging goods or intangible resources (such as knowledge, experience, and time) that individuals own but do not use. As the sharing economic economy market grows, the number of users is also increasing, but many problems arise in terms of reliability between providers and users in the processing of services [3].

The existing shared economic system is a system that requires trust only for the objects shared by the provider and the user. Because of this structure, users suffered economic damages and dissatisfaction by using uncertain suppliers and services. In addition, providers experience such economic losses and service dissatisfaction. In order to activate the sharing economy, it is necessary to confirm mutual reliability between those providing the object to be shared and those using it.

In this paper, we developed a system to establish mutual trust between providers and users in a shared economic environment. In addition, a user measurement system based on a CPS structure in a sharing economic environment is implemented through analysis of the factors to be considered when constructing a CPS.

## 2. Technical Background of the CPS

### 2.1. Concept and Development of the CPS

As wireless sensor network (WSN) technology has evolved, machine-to-machine (M2M) technology that intelligently collects information from a variety of devices is being developed. In other words, the IoT is an evolving form of the existing ubiquitous sensor network (USN) and M2M, and it enables communications not only between things and things, but also between things and people, and people and people [4].

Porter and Heppelmann stated that products using IoT technology will develop into four stages: monitoring, control, optimization, and autonomy. Especially, in the ‘autonomous stage’, IoT-related products are automatically executing and functions such as maintenance are performing autonomously [5]. In other words, IoT technology needs to intelligently perform sensing, networking, and information processing on the distributed-environment elements of sensing technology, communications technology, and service technology under mutual cooperation, without user intervention.

**Figure 1:**
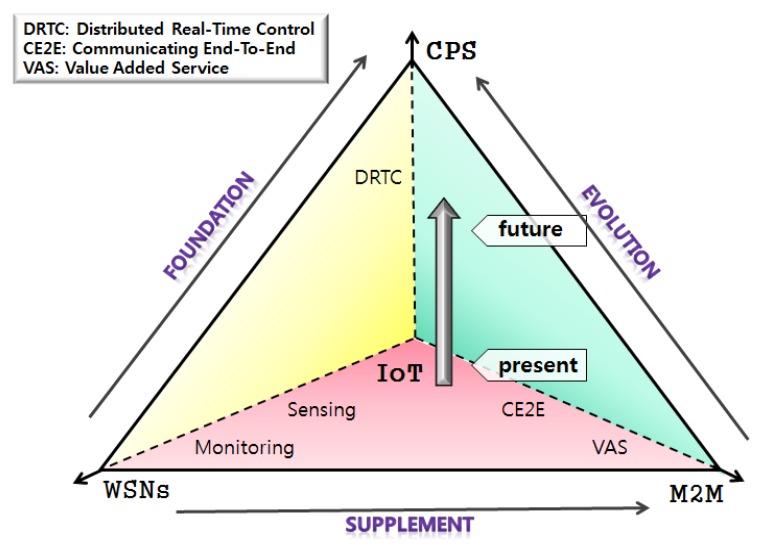
Application range of the CPS.

A CPS is a control system that connects the cyber world with the physical world to merge and analyze real and cyber information, and feed the analyzed data back to the real world. A CPS can control the movement of reality by using data processing results in the cyber world without human intervention. Therefore, CPS is a technology paradigm that reflects IoT technology. In addition, CPS is being studied to enhance utilization in various fields, and it is expected that the social system will be greatly changed through application of CPS systems [6]. Figure 1 shows the evolution and correlation among WSNs, M2M technology, the IoT, and the CPS. Moreover, it shows that advancements in M2M and WSNs are expected to increase the number of CPS applications in the future.

In this paper, we implement a user measurement system based on a CPS structure to improve reliability in a shared economic environment. The system can collect and analyze user context information that changes in real time, and control permissions according to context. In addition, if the user is inappropriate, the provider may deny the user the right to use the shared object.

### 2.2. Dynamic Attributes of Context Information

Context-aware computing is a core technology for providing context-appropriate services to users after processing, such as recognition, analysis, and reasoning about context information collected through sensors. A context is a situation or fact that forms the background of an event, claim or accident [7]. Perera, et al. categorized the situation from two perspectives (‘conceptual view’ and ‘operational view’) for in-depth analysis of the life cycle and tried to recognize the contextual awareness in terms of IoT. In a CPS, dynamic properties of context information must be considered in order to process information exchanged between objects and persons [8].

**Figure 2:**
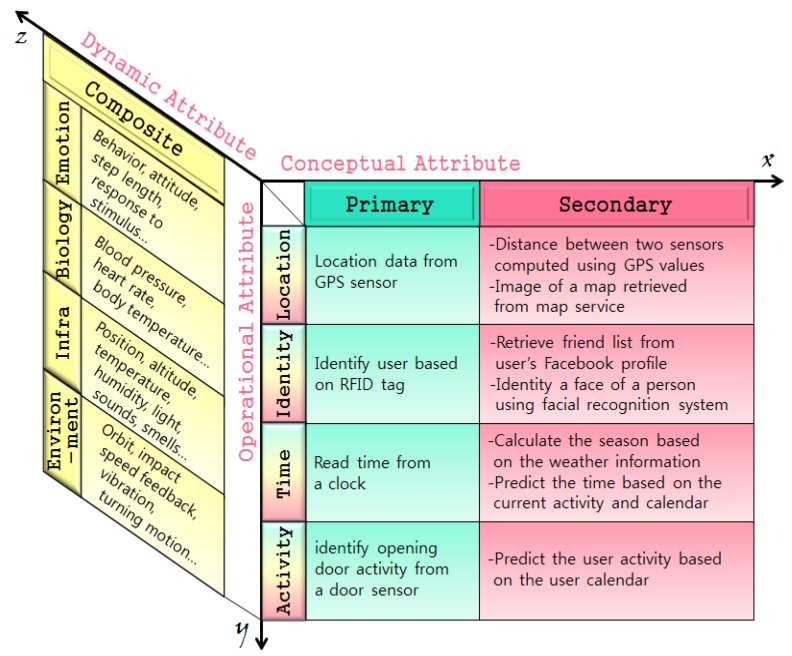
Three-dimensional context classification scheme.

Song, et al. classified context from the viewpoint of the three-dimensions of the *x*, *y*, and *z* axes by including a ‘composite context’ having the attributes of dynamic context information as shown in Figure 2. They suggested that contextual information has dynamic attributes that change continuously, such as emotional or biometric information [9].

The system proposed in this paper is a system that measures entities based on context information gathered from providers, users and shared objects in a shared economic environment, determines contexts and helps decision-making. Therefore, the data that are sensed in the shared object, as well as for the providers and users participating in the shared economic environment can include dynamic attributes. In addition, it can control not only unauthorized users’ usage rights from the provider’s point of view, but also existing shared economic systems that can control user rights and the use of shared objects.

If the shared object is a vehicle, the provider should be able to see the location and the driving record of the vehicle, and the user should be able to see the location, time availability, etc. of the vehicle. In the case of the passenger car sharing system, it is possible to provide personal identification information that can determine the user’s ability to operate by measuring blood pressure, diabetes, alcohol concentration, etc. to determine the user’s reliability [10]. Since the data that are sensed in the shared economic environment contain dynamic attributes, the system design and implementation should consider this.

### 2.3. Varying Context Control from Multi Users and Things

This paper proposes a sharing economy system based on a CPS. In the sharing economy system, dynamic contexts are generating from various objects and various people, so the system’s server should be able to interpret and process a variety of collected information. In a CPS environment, since both people and objects are connected and controlled, it is necessary to form a network of multiple connections beyond two directions. The network includes sensors of various objects and devices of the user, and the context information generated by the user can be process as information for another user.

**Figure 3:**
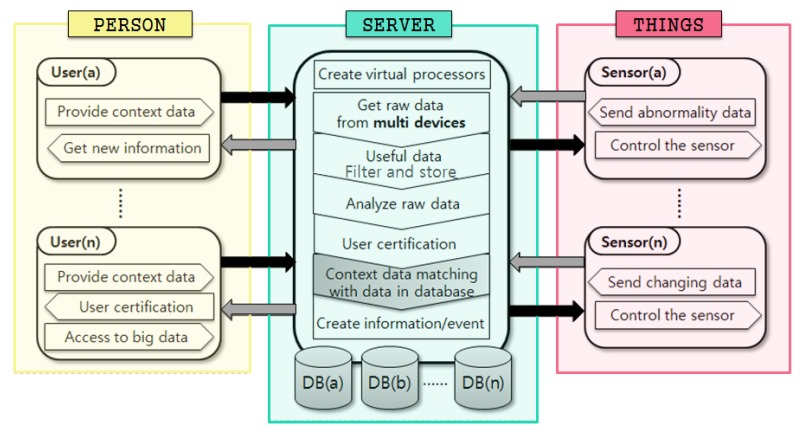
Flow diagram for the contextual information system.

Seo and Kim presented a system structure for multiple-context processing, as shown in Figure 3. In this structure, it is possible to improve the accuracy of context meaning because they are processed as new information through matching between the context information collected from various sensors. Applying the proposed system to a shared economic system, we can define all participants in the shared economic system as users (n). Assuming that the provider is user (a) and the user is user (b), n sensors can be attach to a car to be shared by the provider. User (n) and sensor (n) should support connections between many devices in sharing economy as devices for gathering context information. In addition, these sensor devices can generate real-time data that continuously changes due to characteristics of mobility [11].

Since the amount of the collected data is large, system overload due to data concentration may occur. This causes a variety of problems, such as system and network occurs errors and costs to solve the problems, and makes it impossible to provide a real-time service based on the user context. Therefore, various studies to reduce the system load should be conducted, and it is important to design an optimized system considering interworking between multiple users and multiple sensors.

## 3. Sharing Economy

### 3.1. Issues of Sharing Economy

The sharing economy refers to economic activities in which mutual benefits and appropriate profits can be obtain by mutual lending or exchange of goods or intangible resources, such as knowledge, experience, and time, which are owned by individuals but not used by individuals. As a core element of a sharing economy, an appropriate market for consumer satisfaction should be formed, and the existence of idle assets that can be rented and exchanged mutually is required, with the belief that benefits can be gained through resource sharing. Mutual trust between trading partners is important [12].

**Figure 4:**
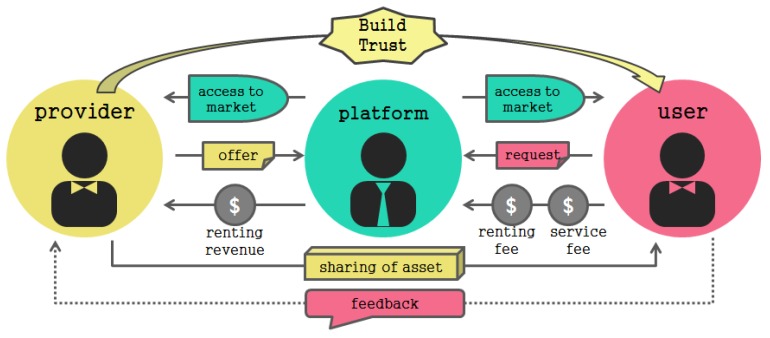
The structure of a sharing economy platform.

Recently, as the shared economic market has grown, the number of users has increased greatly. In order to activate the sharing economy, the provider must also verify the credibility of the user who will use the assets. However, currently the shared economic platform is structured to require reliability only for the objects that the provider and the user share, as shown in Figure 4. Many problems arise in the process of distributing services from the perspectives of uncertainty and mutual reliability between provider and user [13].

A number of incidents with services such as Airbnb and Uber, which are representative sharing service providers, have been reported through the media. In the beds of borrowed houses worms, dirt an even a dead body have been found, and deaths due to carbon monoxide poisoning while sleeping, as well as incidents of house arrest and sexual assault of the guests have occurred. Conversely, the damage from the provider side is also huge, for example, when a house was damaged, valuables were stolen, or an accident caused by a borrowed vehicle by a drunk driver causes a loss. Like this, the shared economy service may cause damage or damaged to the enterprise or user due to a reliability problem of the user. Because the shared economy is distributed based on mutual trust between users and suppliers, there must be a reliable service platform [14]. In other words, it is necessary to develop a system and service platform that can secure mutual reliability between providers and users in a shared economic environment.

Meanwhile, shared economy services are experiencing difficulties in expanding the market due to legal restrictions. In case of South Korea, the Ministry of Strategy and Finance is making efforts to alleviate legal regulations by announcing measures to revitalize the shared economy, in February 2016 [15]. The government has decided to introduce a regulated free zone in the region that applied for the tourism industry as the regional strategic industry, and will introduce it first, and it plans to expand it nationwide after the future progress.

In addition, the Ministry of Strategy and Finance has established various policies to help revitalize the vehicle sharing business. Current vehicle sharing companies can only know whether a member has a license with the license information system of the National Police Agency and the Road Traffic Corporation, and cannot know the type of license and whether the license is suspended. The Ministry of Strategy and Finance has laid out a plan to solve this problem by providing a legal basis for making this information available. Thus, it is possible to inquire about the drunk driving history of a driver that could not be determined before.

In this paper, we propose and implement a shared economic system to solve problems that may occur in the existing shared economy and to increase the reliability of participants in economic sharing. The shared economic structure proposed in this paper can increase the mutual trust among all members (providers, users, etc.) participating in the shared economic environment and can realize the sharing of resources and services. It can also reduce the economic and psychological losses that can occur in the distribution process and increase the satisfaction of all system participants.

### 3.2. Sharing Economy in the IoT Environment

**Figure 5:**
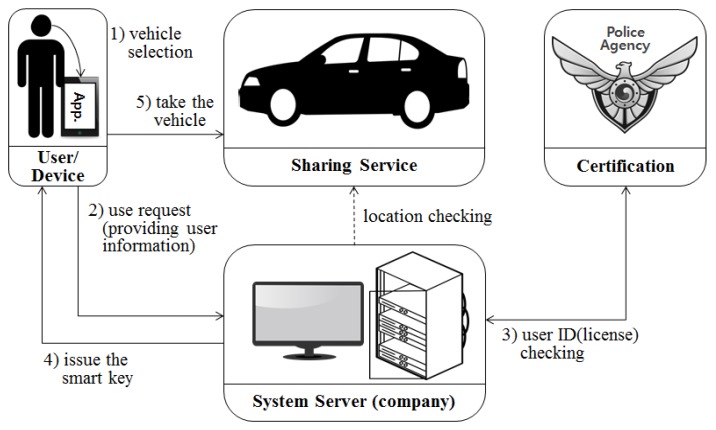
Existing shared vehicle system structure in the IoT.

The existing sharing economy system in the IoT environment has the structure shown in Figure 5. In a vehicle sharing system, companies themselves can provide a shared object (the vehicle) as a service provider, or can provide a service linked to information about a shared object that can be borrowed by a user by contacting various providers.

The user selects a desired time, vehicle type, place, and the like through his/her terminal application, and makes a use request to the enterprise. The enterprise that receives the request from the user checks the user’s license. User licenses are checked only at the time of membership registration, and they are check for whether they match the information registered with the appropriate license issuing body. The server of the enterprise checks the authentication status of the user and issues a smart key to the user’s application. The user can use the service by providing the smart key transmitted to his/her device.

**Figure 6:**
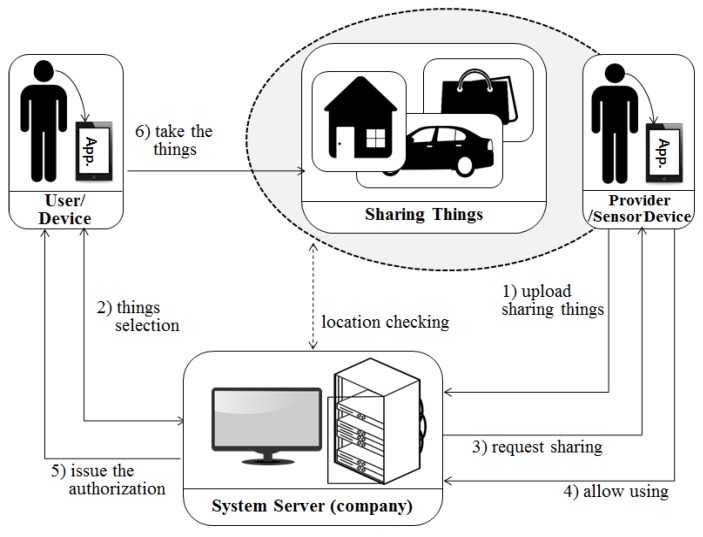
Structure of an existing house sharing system.

In Figure 6, an enterprise sends and receives information about a shared object (house) between a user and a provider, and only provides a platform for real sharing. The users are divided into a provider who wants to provide a house according to the service request purpose and a general user who wants to rent a house. The way a general user requests a service is similar to a vehicle sharing system. The provider uses his/her terminal application to upload information about the ‘house’. After a transaction make with user, the provider can pay the company a commission and receive a rental fee from the user.

In the existing structure, it is not possible to judge the context information of the user, and since user authentication is checked only when the service is first used, it is not possible to discriminate a user who is qualified to use the shared object. For example, when a user wants to use a shared vehicle while drinking alcohol, the system provides the license without any restriction.

In this paper, we present a system that can determine the reliability of users, which is a problem of existing shared economic system. The proposed system is a CPS system that supports reliability measurement of the members based on the user’s context information, and it can control the user’s behavior according to the result.

## 4. Design of the CPS System in a Sharing Economy Environment

### 4.1. System Stucture

**Figure 7:**
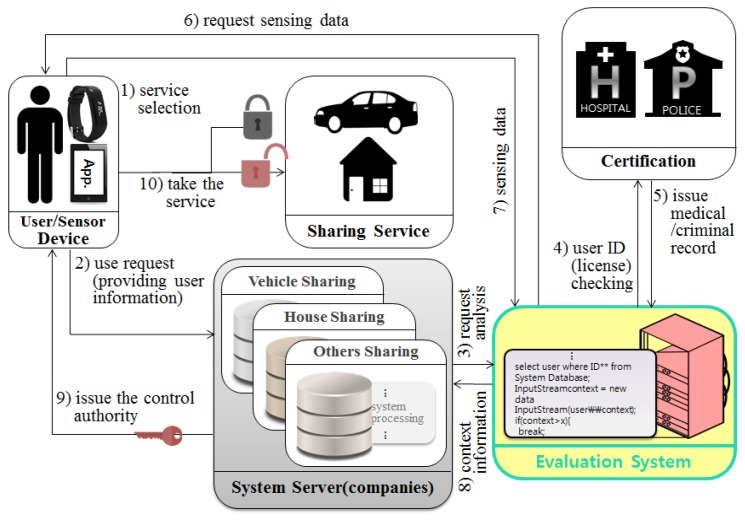
CPS structure for measuring a user’s reliability.

**Figure 8:**
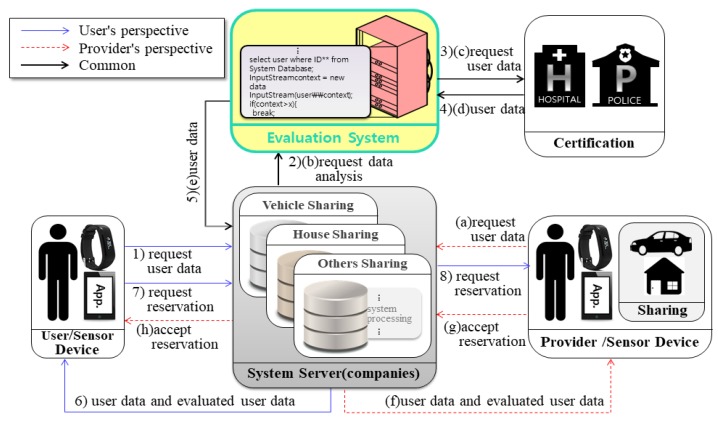
Information analysis flow diagram for providers and users.

**Figure 9:**
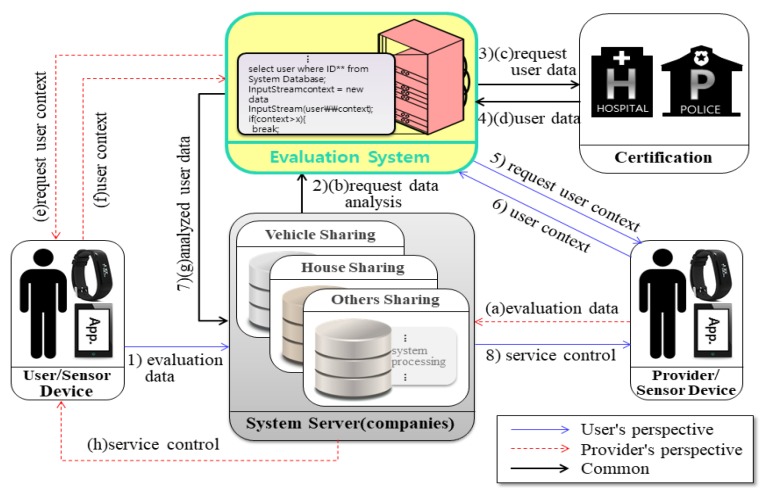
The evaluation system flow for service control.

The system presented in this paper can divided into three perspectives. Figure 7 shows the system for extended user reliability measurement based on Figure 5 of the existing system. It shows the CPS structure for measuring the user’s reliability using the user’s context awareness information. Figure 8 and Figure 9 in this paper proposed extended design based on the existing system in Figure 6. Figure 8 shows the process of requesting the analysis of the information of the transaction object for the quality reliability of the shared object before using the shared economic service. Figure 9 shows the measurement system structure between the provider and the user for controlling the user’s usage rights after using the shared economic service.

#### 4.1.1. Reliability Measurement System Based on User’s Context Awareness Information

Figure 7 shows the CPS structure for measuring the user’s credibility using the user’s context-aware information. The proposed system collects and analyzes context information about users (which changes in real time) and can control the usage rights according to the context as with Table 1. For example, if a user requests to use a shared vehicle after drinking, the system determines that the user has drunk using the collected sensor data values, and then he/she cannot use the shared vehicle if he or she drank.

#### 4.1.2. Analyzing User and Provider Information to Determine the Quality of Shared Objects

Figure 8 shows the process of requesting information analysis between a user and a provider to judge the quality of a shared object before using the shared economic service.

Steps (1) to (8) are the information analysis flow from the user’s perspective of view, and (a) to (h) are the information analysis flow from the provider perspective. In the system flow, we represented duplicated parts as one. The measured user evaluation information can improve the reliability between users and providers in the shared economic environment. Table 2 describes the method of analyzing the provider and the user for determining the quality of the shared object.

#### 4.1.3. Evaluation between Provider and User for Continuous Control of Service Use.

Figure 9 shows the post-evaluation system structure for establishing trust between the provider and the user. The provider and the user evaluate each other after using the shared object, and the company’s system analyzes the evaluation contents based on the situation information. The system controls the user’s platform usage based on the analyzed information, thereby improving the trust between the user and the provider. Steps (1) to (8) is a flow in which a user evaluates a provider by using a shared object, and (a) to (h) is a flow in which a provider evaluates a user. In the system flow, we represented duplicated parts as one. Table 3 describes the flow in which the user evaluates the provider after using the share and the other flow in which the provider evaluates the user.

### 4.2. Data Processing Module

**Figure 10:**
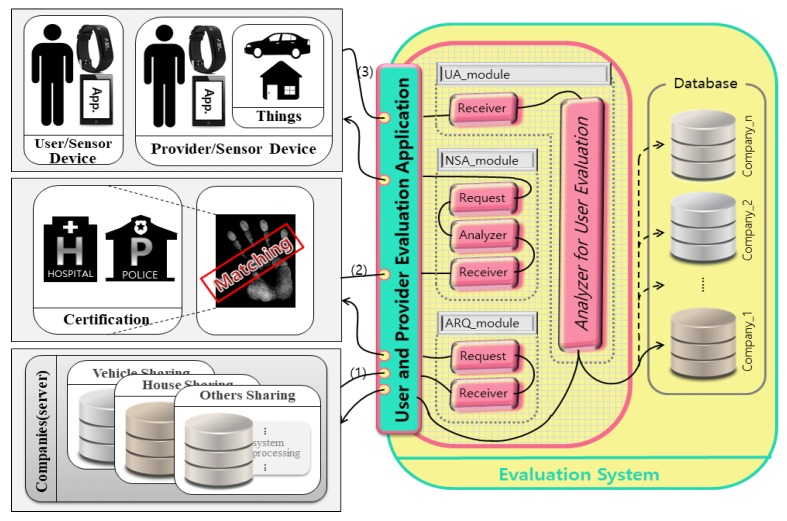
Internal structure of the user’s reliability measurement system.

The internal structure of the user’s reliability measurement system proposed in this paper is show in Figure 10. The *User Measurement Application* consists of a User Analysis_module (UA_module) for user analysis, Necessary Sensors Analysis_module (NSA_module) for required sensor analysis, and an Analysis Request_module (ARQ_module) for requesting analysis.

The NSA_module requests user an analysis from the *Receiver* of the evaluation application in the company’s server according to flow (1), and requests the user’s criminal and high level health information for certification through the request. NSA-module sends the requested information to the *Receiver* of the *Evaluation Application* according to flow (2). The *Analyzer* works with real-time sensing data required for user analysis based on the transmitted information. It obtains real-time sensing data from the user through *Requests*. UA-module receives the requested sensing data from the provider and user according to the flow (3) and analyzes the current context of the user based on the received sensing data in Analyzer for User Evaluation. It then stores the analyzed information in the corresponding company’s database and sends it to the company’s server.

**Figure 11:**
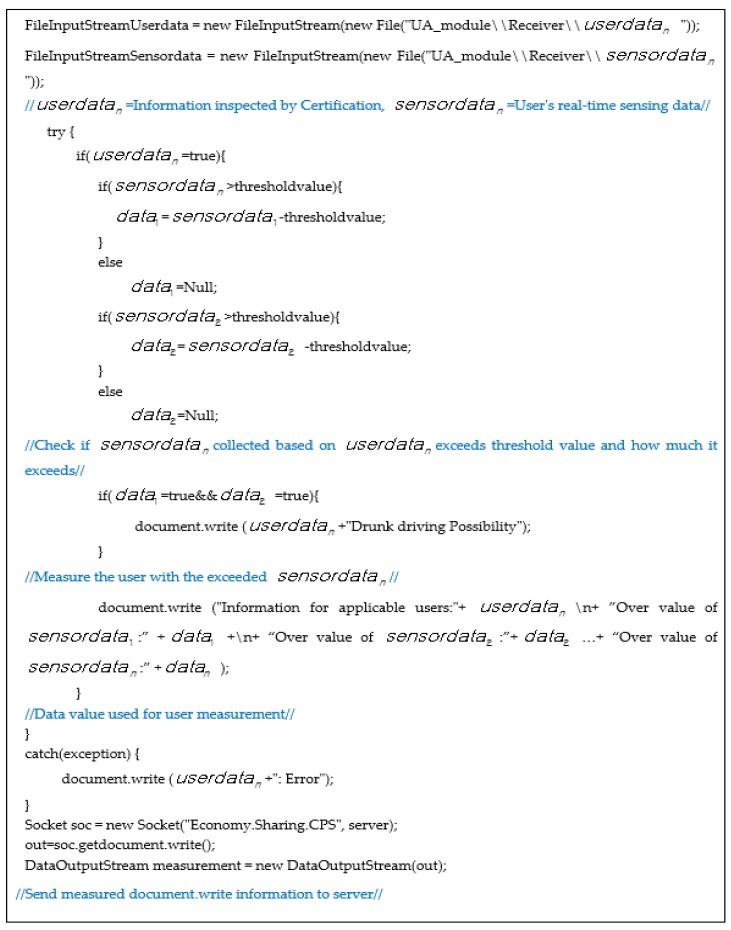
Design algorithm of the system.

Figure 11 shows an algorithm that collects and analyzes user contextual information in the sharing economy environment and determines the reliability of users who use the shared objects. *userdata_n_* is the information received by the certification, and *sensordata_n_* is the user’s real-time sensing data. It checks whether the value collected based on *userdata_n_* exceeds the threshold value and how much it is, and then measures the user by using the exceeded *sensordata_n_* value, and transmits the measured document.write information to the server.

**Figure 12:**
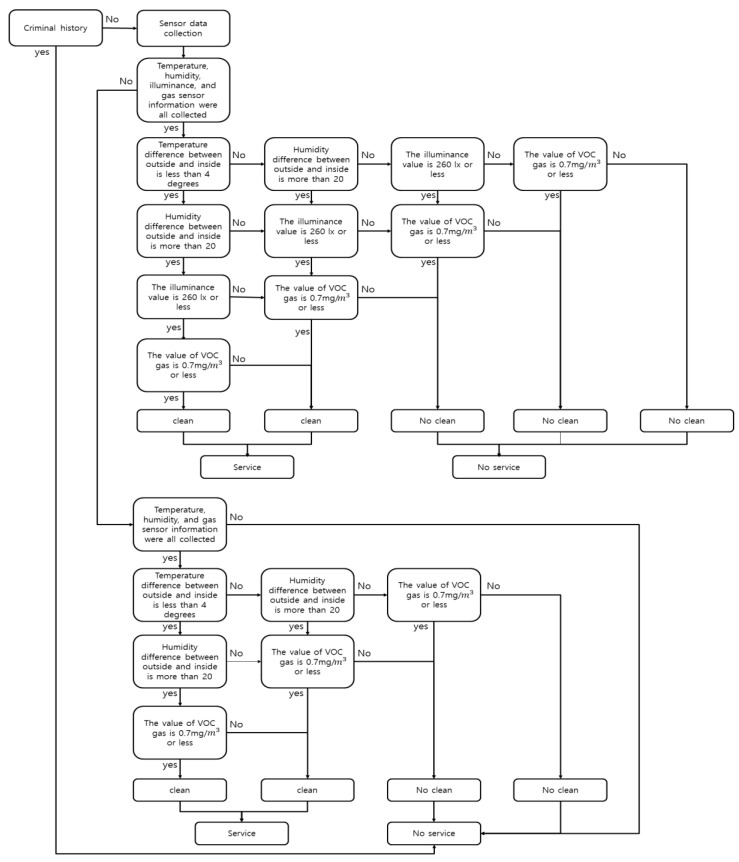
Flow diagram for processing sensor data.

Figure 12 shows a model that analyzes the context based on the above structure using the input data and derives the command. In order to analyze the comfortable environment condition of the shared object, the difference between the outdoor temperature and humidity data and the indoor temperature and humidity data values is used. It is the illumination and the gas data of the input data using only the data sensed indoors for the context analysis. The four sensed data used in the context analysis can determine whether the shared object is comfortable, and finally decide whether to provide the service or not.

## 5. Implementation of the CPS in a Sharing Economy Environment

### 5.1. System Implementation

**Figure 13:**
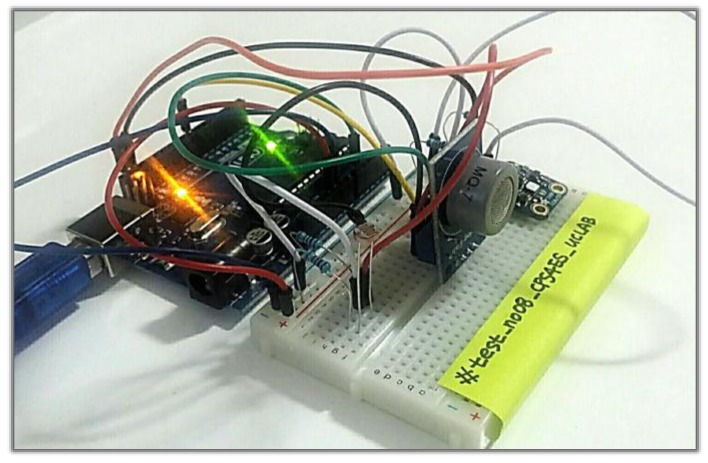
The Arduino used in the system implementation.

We have implemented the system designed in this paper and performed a simulation test. To simulate the proposed system, we assumed a “shared house” environment and used four sensors (temperature, humidity, lighting and volatile organic compounds (VOCs)) to determine whether the accommodation was clean. For the system implementation, we also coded using the Arduino sketch program of the Arduino version 1.8.3 and with XML and Java using Android Studio version 2.3.2. Figure 13 shows the Arduino used in the system implementation.

**Figure 14:**
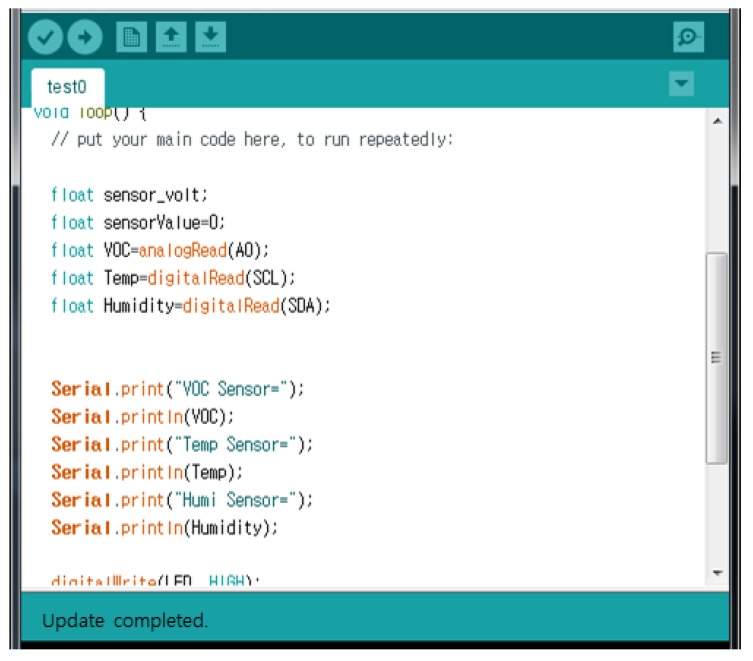
Coding screen for obtain sensing data using Arduino.

Figure 14 is a screen in which temperature, humidity, illumination, and VOC gas for odor sensing is coded at intervals of 3 s for cleanliness context information of the user’s room. It uses the Arduino to operate the sensor and transmit the sensed data values.

**Figure 15:**
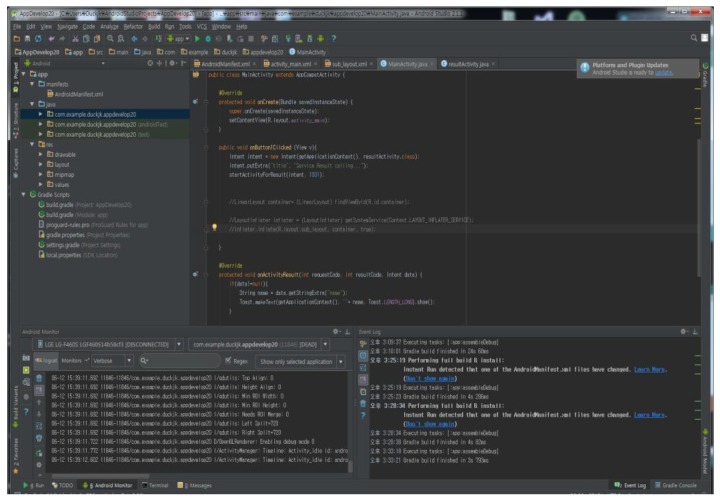
Coding screen provide the service after analyzing the sensed values in Arduino.

Figure 15 shows that Arduino is coded to provide the service after analysis through Android Studio. First, we check the temperature, humidity, light and VOC gas sensing values that can be measured for cleanliness. If the temperature is high but the humidity is low, the context inferred is that there is no problem with cleanliness, and if the temperature is high and the humidity is high, an alarm is generated that attention should be paid to the cleanliness. In addition, if the temperature and humidity are not appropriate, and VOC gas is high, it is judge that the state of the ‘shared room’ is not clean. We coded to stop the service if the system receives this result.

### 5.2. Simulation Results

**Figure 16:**
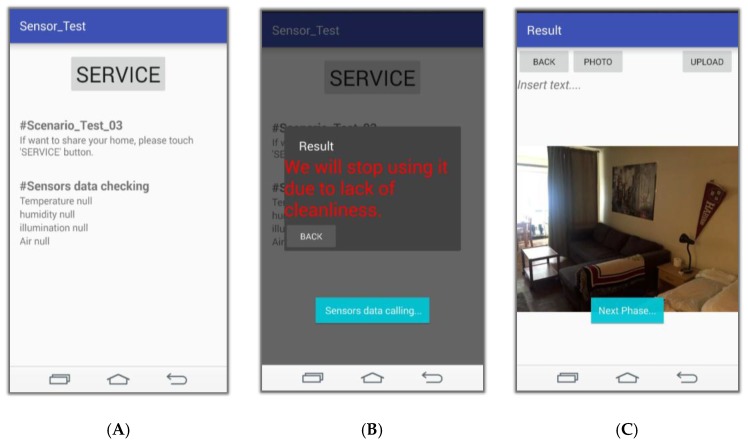
Screen for the service application: (**A**) Application access and Confirm sensing value; (**B**) Non-conformity after service execution; (**C**) Enter information about the share target.

This section presents the simulation results. Figure 16 shows the application of the service. Figure 16A shows the screen in which the user accesses the application to use the service. Figure 16B indicates that the cleanliness of the ‘shared house’ is bad due to the sensed data value when the service is executed by pressing the ‘SERVICE’ button, and this screen rejects the service. Figure 16C is a screen for providing the service because the sensed data value is inferred to be clean when the ‘SERVICE’ button is pressed. Users can upload photos and descriptions of the objects they want to share on this page.

## 6. Conclusions

In this paper, we have developed a system to establish mutual trust between providers and users in a sharing economic environment. In order to implement a system that can measure and control users’ situation in a shared economic environment, we analyzed the necessary factors in CPS. In addition, a user measurement system based on a CPS structure in a sharing economic environment is implemented through analysis of the factors to consider construction of a CPS. In particular the existing methods that have been provided for shared economic systems judge the reliability of the provider from the viewpoint of the user. In this paper, we have implemented a system that can also judge the user’s reliability for the provider, that is, a system to judge the authority control of users’ shared services from the provider’s perspective through the user’s context information in the system. The system proposed in this paper is applicable to various fields such as culture, life, health, education, traffic. Therefore, it will be used to expand various CPS researches and services.

In the future, we will confirm whether the indicator of user’s reliability apply quantitatively by actually applying it to a shared economic environment. Since the proposed system controls the system using personal information such as user’s context information, the security of the system should improve. Therefore, the security problems that may arise when operating with sensitive information received from a certification authority should be solved.

## Figures and Tables

**Table 1:** Procedure for measuring user’s reliability.

Phase	Contents
Step (1)	Users select smart devices to give the information of when, where, and what services to use.
Step (2)	The user requests the server to give permission to use the selected service.
Step (3)	The server requests the user evaluation system to analyze the user who requested the use of the service.
Step (4)	The user measurement system requests the certification body to provide the information such as the past criminal history and hospital record of the user who requested the use of the service.
Step (5)	The certification body sends the relevant information to the user measurement system.
Step (6)	The user measurement system is request to user real-time sensing data based on the information received from the certification authority (ex. requesting current blood alcohol concentration data from wearable device if the user has the record of drunken driving).
Step (7)	Collecting and sending user’s sensing data.
Step (8)	The user measurement system analysis the user’s information received from the certification authority and the real-time sensing data sensed by the user, and send to server.
Step (9)	The server provides control (smart key) after determining whether to provide the service requested by the user based on the user’s context information.
Step (10)	The user can use the service by using the control (smart key).

**Table 2:** Procedure for judging the quality of the shared object in the system.

Evaluates of User’s Perspective		Evaluates of Provider’s Perspective	
Step (1)	Request the provider information of the sharing service selected by the user to the server of the enterprise.	Step (a)	The provider asks the company server for the information of the user who made the reservation application.
Step (2)	The server of the enterprise requests the information of the provider to the user evaluation system to be developed.	Step (b)	The company’s server requests the user’s evaluation system for the user’s information.
Step (3)	The user evaluation system requests the provider’s information from the certification authority.	Step (c)	The user evaluation system requests the user’s information from the certification authority.
Step (4)	The certification provides the user evaluation system with the information of the provider.	Step (d)	The certification provides the user evaluation system with the information of the provider.
Step (5)	The user evaluation system provides the server with the provider information received from the certification.	Step (e)	The user evaluation system provides the server with the user information received from the certification.
Step (6)	The server sends the provider information from the user evaluation system and the evaluation records of the provider evaluated in Figure 9 together to the user.	Step (f)	The server sends the user information received from the user evaluation system and the evaluation records of the user evaluated in Figure 9 to the provider.
Step (7)	The user browses all the information of the provider from the company’s server and requests the server to the reservation.	Step (g)	The provider reads all the information of the user received from the company and sends a reservation approval to the company’s server.
Step (8)	The company’s server notifies the user’s reservation request to the provider.	Step (h)	The company’s server checks the approved information and notifies the user that the reservation is approved.

**Table 3:** Process of evaluation between providers and users in the system.

Evaluates of User’s Perspective		Evaluates of Provider’s Perspective	
Step (1)	The user inputs the evaluation information about the provider after using the shared economic service.	Step (a)	The provider inputs the evaluation information about the user after using the shared economic service.
Step (2)	The company’s server requests the developed user evaluation system to analyze the provider evaluation information.	Step (b)	The company’s server requests the developed user evaluation system to analyze the user evaluation information.
Step (3)	The user evaluation system asks the certification authority for information about the provider.	Step (c)	The user evaluation system asks the certification authority for information about the user.
Step (4)	The certification authority provides the provider information (such as criminal history) to the user evaluation system.	Step (d)	The certification authority provides the user information (such as criminal history) to the user evaluation system.
Step (5)	The user evaluation system requests sensing data from the provider.	Step (e)	The user evaluation system requests sensing data from the user.
Step (6)	The user evaluation system collects the provider’s sensing data.	Step (f)	The user evaluation system collects the user’s sensing data.
Step (7)	The user evaluation system sends to the company server the provider’s information received and the sensing data analyzed from the certification authority.	Step (g)	The user evaluation system sends to the company server the user’s information received and the sensing data analyzed from the certification authority.
Step (8)	The company’s server decides whether to provide the platform service continuously to the provider based on the analyzed context information.	Step (h)	The company’s server decides whether to provide the platform service continuously to the user based on the analyzed context information.
